# Extensive Macular Atrophy With Pseudodrusen Complicated by Macular Neovascularization in a Japanese Patient: A Case Report

**DOI:** 10.7759/cureus.93258

**Published:** 2025-09-26

**Authors:** Taro Kominami, Junya Ota, Jun Takeuchi, Kenya Yuki, Hiroaki Ushida

**Affiliations:** 1 Ophthalmology, Nagoya University, Nagoya, JPN

**Keywords:** anti-vegf therapy, extensive macular atrophy with a pseudodrusen-like appearance, japanese, macular neovascularization, retina

## Abstract

Recognizing the characteristic vertically oriented atrophy, pseudodrusen distribution, and retinal pigment epithelium-Bruch's membrane separation is critical for distinguishing extensive macular atrophy with a pseudodrusen-like appearance (EMAP) from age-related macular degeneration (AMD). Early identification of neovascular complications and prompt anti-vascular endothelial growth factor therapy can stabilize macular neovascularization (MNV) and help preserve residual vision in this rare retinal disorder. To the best of our knowledge, this is the first reported case of EMAP with MNV in an Asian patient. This report aims to describe the clinical presentation, imaging features, genetic findings, and therapeutic response in a Japanese woman with EMAP complicated by MNV, which is rarely reported in Asia. A 63-year-old woman presented with decades-long nyctalopia and progressive visual loss. Fundus examination and fundus autofluorescence showed vertically oriented macular atrophy, widespread pseudodrusen, and peripheral paving-stone degeneration. Optical coherence tomography (OCT) demonstrated diffuse separation of the retinal pigment epithelium from Bruch's membrane. These findings lead to the diagnosis of EMAP. Fluorescein angiography, indocyanine green angiography, and OCT angiography revealed type 1 MNV in the left eye. Whole-exome sequencing detected no pathogenic variants associated with inherited retinal disease or AMD. The neovascular lesion was treated with intravitreal aflibercept on a treat-and-extend regimen; after seven injections, the MNV became inactive.

## Introduction

Extensive macular atrophy with a pseudodrusen-like appearance (EMAP) is a rare and rapidly progressive retinal disease that was first described in France in 2009 [1]. EMAP was originally characterized by symmetrical and vertically oriented macular atrophy with multilobular borders, widespread pseudodrusen-like deposits from the posterior pole to the mid-peripheral retina, and far peripheral retinal paving stone degeneration in both eyes. EMAP frequently begins in middle-aged individuals, typically under 55 years of age, and progresses to severe visual impairment [2-4]. Early symptoms typically include night blindness (nyctalopia), photophobia, and vision loss [1,2].

EMAP presents diagnostic challenges because of its overlap with age-related macular degeneration (AMD). However, features such as nyctalopia, earlier onset, peripheral lesions, characteristic vertical expansion of atrophy, and lack of foveal sparing at an early age help differentiate EMAP from AMD [1]. Advanced imaging modalities, particularly optical coherence tomography (OCT) and short-wavelength fundus autofluorescence (SW-AF), are instrumental in identifying EMAP-specific features. OCT reveals the hallmark diffuse separation between the retinal pigment epithelium (RPE) and Bruch’s membrane, whereas SW-AF shows areas of moderately decreased autofluorescence unique to this condition. Interestingly, genetic variants typically linked to inherited retinal dystrophy (IRD) such as Stargardt disease are absent in most cases of EMAP, indicating a multifactorial or environmental origin [3,5,6]. Recent studies have also highlighted the utility of multimodal imaging in disease staging [4].

Despite its increasing recognition in Europe, EMAP remains a "niche diagnosis," which is often underreported or misdiagnosed, particularly in non-European populations. It is commonly misclassified as AMD, especially among patients older than 60 years, due to shared imaging features such as pseudodrusen deposits and macular atrophy, such as the trickling pattern of geographic atrophy [7]. However, it exhibits distinct differences, including a younger age of onset, faster progression, and specific multimodal imaging findings. Most documented cases have been concentrated in France and Italy, where EMAP clusters have been observed, suggesting regional environmental or occupational risk factors [3,5,8,9]. Outside Europe, cases have been sporadic, with 18 reported cases in Brazil [6] and only one case reported to date in Japan [10]. This underscores the need for greater awareness and accurate diagnosis, particularly in Asian populations.

Another layer of complexity is the occurrence of macular neovascularization (MNV) in patients with EMAP. EMAP was originally reported not to be associated with choroidal neovascularization (CNV) [1], but some EMAP cases have been accompanied by CNV [11-13]. MNV presents as a potentially blinding complication that mimics neovascular AMD in its aggressiveness. Anti-vascular endothelial growth factor (anti-VEGF) therapy, commonly used in AMD, has shown promise for controlling MNV activity in patients with EMAP, although long-term outcomes remain underexplored.

Here, we present the case of a Japanese woman diagnosed with EMAP complicated by MNV. After conducting a literature review on July 20, 2025, utilizing PubMed and Google Scholar, and using the keywords "extensive macular atrophy with pseudodrusen" and "neovascularization", we did not find any prior reports of Japanese EMAP cases, except one by Sato et al. [10], and of MNV. This case is clinically significant not only because it is the first report of this complication in an Asian patient, but also because it carries a powerful message for clinicians worldwide. In regions where EMAP is a "niche diagnosis," it is essential that clinicians consider it in the differential diagnosis of progressive macular atrophy to avoid misclassification as AMD. Furthermore, this report highlights a crucial and hopeful point: while EMAP itself leads to severe vision loss, its neovascular complications are treatable. Our findings provide clear evidence that timely anti-VEGF intervention can successfully stabilize MNV, offering a practical strategy to preserve remaining vision in patients with this devastating rare disease.

## Case presentation

The patient is a 63-year-old Japanese woman. She had first presented at the age of 49 years with visual field defects detected during a routine health examination. Her primary complaint was progressive visual impairment, accompanied by a significant history of night blindness (nyctalopia), which she had experienced for many years. Despite the treatment of suspected glaucoma with prostaglandin eye drops in previous clinics, further evaluation revealed no glaucomatous optic neuropathy. Cranial CT ruled out intracranial disease. At the initial examination, the best-corrected visual acuity was 20/40 in the right eye and 20/32 in the left eye.

The patient had no family history of IRD, glaucoma, or AMD. There was no occupational history of toxic exposure, particularly during farm work. Her systemic medical history included hyperlipidemia, renal cysts, colonic polyps (treated endoscopically), and oral lichen planus. She had also received hormonal therapy for menopausal symptoms in the past, but had no inflammatory history. She had no history of ischemic heart disease. Based on the findings of macular degeneration, an IRD specialist initially diagnosed the patient with macular dystrophy, but whole exome sequencing revealed no pathogenic variants typically associated with IRDs and AMDs, including *TIMP3*, *C1QTNF5*, *ARMS2*, and *HTRA1* [14].

**Figure 1:**
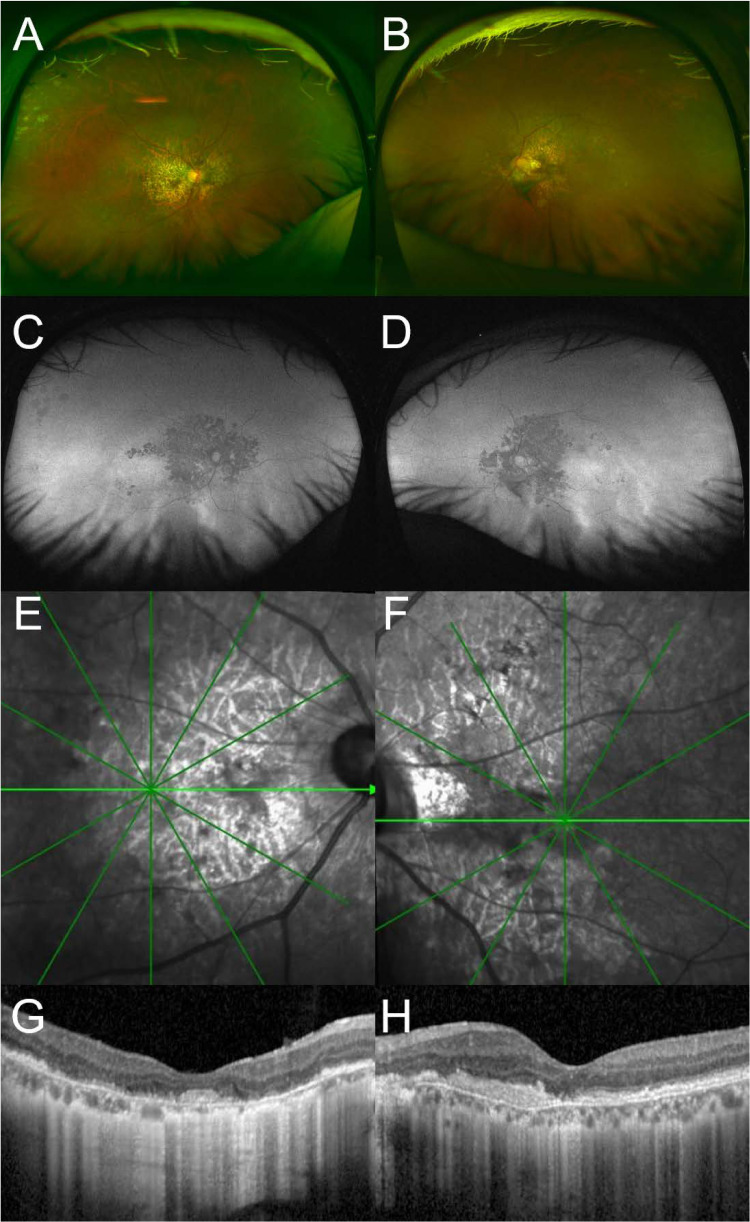
Multimodal imaging findings in a patient with advanced retinal degeneration at a follow-up visit at 63 years of age (A, B) Widefield pseudocolor fundus photographs of the right (A) and left (B) eyes show macular degeneration with pseudodrusen extending from the posterior pole to the mid-peripheral region. In the right eye, extensive paving stone degeneration is evident in the far peripheral retina. (C, D) Widefield fundus autofluorescence (FAF) images of the right (C) and left (D) eyes reveal vertically elongated hypoautofluorescent areas corresponding to macular degeneration. The hypoautofluorescent zones extend nasally, exhibiting widespread involvement. (E, F) Infrared reflectance images of the right (E) and left (F) eyes provide complementary visualization of macular abnormalities. (G, H) Optical coherence tomography (OCT) horizontal scans of the right (G) and left (H) eyes depict structural changes suggestive of fibrosis, consistent with Stage 3 degeneration in both eyes.

During the present follow-up, at 63 years of age, the patient exhibited progressive macular changes characterized by vertically oriented atrophy of the posterior pole (Figure 1A, 1B). Fundoscopic findings revealed pseudodrusen deposits distributed across the macula and mid-periphery as well as peripheral paving stone-like degeneration in the right eye (Figure 1A). Wide-field fundus autofluorescence (FAF) images showed a central hypoautofluorescent area with an intact temporal region (temporal sparing), consistent with EMAP (Figure 1C, 1D). Infrared macular images revealed macular atrophy with a large vertical axis (Figure 1E, 1F). OCT revealed fibrosis in both eyes (Figure 1G, 1H), indicating stage 3 EMAP [4], and identified a separation between the RPE and Bruch’s membrane in the left eye, which is a hallmark of EMAP (Figure 1H). Based on these clinical findings, the diagnosis was revised to EMAP, rather than macular dystrophy.

**Figure 2:**
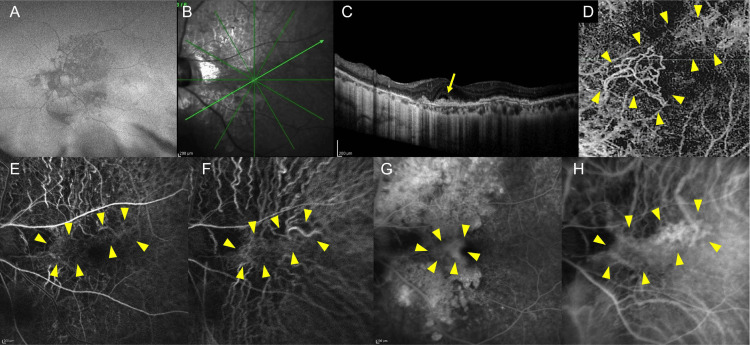
Multimodal imaging findings of the left eye at the onset of macular neovascularization (MNV) at the age of 59 years (A) Widefield fundus autofluorescence image shows vertically elongated hypoautofluorescent areas consistent with macular degeneration, in agreement with the findings at the final visit. (B) Infrared fundus image highlights the region of interest for subsequent imaging modalities. (C) Optical coherence tomography (OCT) oblique scan, corresponding to the section indicated by the dark green arrow in (B), demonstrates subretinal fluid accumulation (yellow arrow). (D) OCT-angiography image, using a slab from the outer plexiform layer to the choriocapillaris, reveals a suspected MNV area indicated by the yellow arrowhead. (E) Early-phase fluorescein angiography (FA) image identifies a suspected abnormal vascular network (yellow arrowhead). (F) Early-phase indocyanine green angiography (IA) image shows a suspected abnormal vascular network in the same region (yellow arrowhead). (G) Late-phase FA image reveals leakage in the region marked by the yellow arrowhead, distinct from the hyperfluorescent area due to a window defect. (H) Late-phase IA image confirms leakage in the region marked by the yellow arrowhead, consistent with MNV.

At the age of 59 years, as shown in the wide-field FAF image (Figure 2A) and the infrared macular image (Figure 2B), the OCT of the left eye had suggested the presence of subretinal fluid (Figure 2C). OCT angiography, fluorescein angiography, and indocyanine green angiography revealed MNV in the left eye (Figure 2D-2H).

The patient was then diagnosed with EMAP, a rare retinal condition complicated by MNV of the left eye. The patient received intravitreal aflibercept (2 mg, 0.05 mL) injections in the left eye to treat MNV using a treat-and-extend protocol. The injection intervals were extended to 16 weeks after initial stabilization of the disease. In total, seven injections were administered, after which the treatment was discontinued, and MNV did not recur during follow-up without any adverse events. This regimen effectively controlled neovascular activity and prevented further deterioration of the left eye.

At the last follow-up visit, at 63 years of age, the patient’s visual acuity had declined to 20/2000 in the right eye and 20/100 in the left eye. Although macular atrophy has progressed significantly and may continue with progressive deterioration of visual function, to date, the left eye has remained stable with no recurrence of MNV after completing the aflibercept treatment regimen. The patient adhered well to the planned treat-and-extend regimen with intravitreal aflibercept injections, and no issues with tolerability were reported throughout the course. Adherence was inferred from consistent attendance at scheduled visits and absence of treatment delays.

## Discussion

This case suggests the importance of early detection and proactive management of MNV in patients with EMAP to preserve the remaining vision and prevent further complications. The report highlights two significant findings. First, the diagnosis of EMAP in a Japanese patient, a condition that remains underrecognized in Asian populations, highlights the need for heightened awareness and improved diagnostic approaches in non-European contexts. Second, the development of MNV as a complication of EMAP, which was successfully treated with anti-VEGF therapy, demonstrates the importance of monitoring and managing neovascular complications in this rare condition. These findings provide valuable insights into the understanding and management of EMAP, particularly in regions where it has rarely been reported.

The first key finding of this case is the diagnosis of EMAP in a Japanese patient, marking one of the few documented instances in Asia [10]. EMAP is reported predominantly in European populations, with most cases described in France and Italy [2,3,5,8,9]. Diagnostic challenges arise due to its clinical and imaging overlap with AMD [7]. However, specific features such as nyctalopia, vertically oriented atrophy, and peripheral paving stone-like degeneration distinguish EMAP from AMD. Indications that this case is likely to be EMAP and not AMD include the patient's complaint of night blindness, the onset at a young age of 49 years (assuming that the onset was at the time of the first visit to Nagoya University Hospital), vertically greater diameter degeneration specific to EMAP, paving stone degeneration of the far peripheral retina, and the absence of cardiovascular history which is correlated with trickling pattern of geographic atrophy [15].

The fundus phenotype of EMAP overlaps with the trickling pattern of geographic atrophy in many areas [3,7], but while “EMAP” denotes a diagnosis, “diffuse-trickling” was conceived to describe the pattern of hyper autofluorescence surrounding geographic regions. These findings are further corroborated by advanced imaging techniques such as OCT and fundus autofluorescence, which reveal hallmark features, including diffuse separation of the RPE from the Bruch’s membrane and temporal sparing [4]. This case emphasizes the need for greater recognition of EMAP in Asian populations, where it is often misdiagnosed owing to a lack of awareness. Increased reporting and utilization of multimodal imaging can aid in distinguishing EMAP from other retinal disorders, thereby improving diagnostic accuracy and management strategies.

The second key finding of this case was the development of MNV as a complication of EMAP. Although rare, MNV has been reported in advanced stages of EMAP [11-13] and poses a significant threat to vision if left untreated. In this patient, the MNV was effectively managed with intravitreal aflibercept injections using a treat-and-extend protocol, leading to stabilization without recurrence. This outcome highlights the potential of anti-VEGF therapy in controlling neovascular activity in EMAP, mirroring its established role in the management of neovascular AMD. This case demonstrates the importance of routine monitoring for neovascular complications in patients with EMAP. Although EMAP is primarily characterized by atrophic changes, the emergence of MNV necessitates a prompt diagnosis and treatment to preserve the remaining vision. Furthermore, this finding may contribute to the understanding of MNV pathophysiology in EMAP and highlights the need for further research on its prevalence, progression, and response to therapy.

In addition to providing information for clinical practice, this case highlights the need for expanded research on EMAP in non-European populations. Studies focusing on genetic, environmental, and inflammatory underpinnings may provide insight into regional differences in prevalence and presentation. It should, however, be noted that this report describes a single case of EMAP complicated by MNV, and the findings may not be generalizable to all patients with EMAP.

## Conclusions

This case demonstrates the significance of recognizing EMAP in non-European populations and highlights its potential complication with MNV. The use of multimodal imaging is critical in achieving an accurate diagnosis, whereas anti-VEGF therapy has proven effective in managing neovascular activity and preventing further vision loss in EMAP. This case reinforces the need for increased clinical awareness and ongoing research. The lessons learned from this case can guide clinicians in improving diagnostic accuracy, monitoring complications, and tailoring treatment strategies for this rare and challenging condition.
